# Clean interface without any intermixed state between ultra-thin P3 polymer and CH_3_NH_3_PbI_3_ hybrid perovskite thin film

**DOI:** 10.1038/s41598-019-47252-y

**Published:** 2019-07-26

**Authors:** Min-Cherl Jung, Asuka Matsuyama, Sora Kobori, Inhee Maeng, Young Mi Lee, Myungkwan Song, Sung-Ho Jin, Masakazu Nakamura

**Affiliations:** 1 Division of Materials Science, Nara Institute of Science and Technology, Ikoma, Nara 630-0192 Japan; 2 Advanced Photonics Research Institute, Gwangju Institute of Science and Technology, Gwangju, 61005 Republic of Korea; 3 Beamline department, Pohang Accelerator Laboratory, POSTECH, Pohang, 37673 Republic of Korea; 4 Surface Technology Division, Korea Institute of Materials Science (KIMS), Changwon, Gyeongnam 642-831 Republic of Korea; 5 Department of Chemistry Education Graduate, Department of Chemical Materials, and Institute for Plastic Information and Energy Materials, Pusan National University, Busan, 46241 Republic of Korea

**Keywords:** Materials science, Applied physics

## Abstract

Hole transport layers (HTL) are crucial materials to improve the power conversion efficiency in organohalide hybrid perovskite-based solar-cell applications. Two important physical properties are required in HTL materials: good hole mobility and air-protection. After HTL solution-based deposition, an intermixed chemical state at the interface between HTL and hybrid perovskite is key to confirming the physical property of HTL. We performed high-resolution x-ray photoelectron spectroscopy to investigate the chemical states at the interface between an ultra-thin P3 polymer and CH_3_NH_3_PbI_3_ hybrid perovskite thin film. At the interface, we found no apparent intermixed chemical state. Furthermore, we confirmed that the P3 HTL with the ultra-thin layer (7 nm) protected the hybrid perovskite material against air-exposure for 2 weeks.

## Introduction

Organohalide perovskite (OHP) is one of the great candidate materials for solar-cell application^1,2^. Recently, the power conversion efficiency (PCE) is already over 23.7%, which is now highly competitive with other solar-cell materials. such as CdTe (22.1%), CIGS (22.6%), and Si (26.0%)^2^. After the first report from the Miyasaka group in 2009^3^, researchers have tried many engineering approaches to changing hole transport layers (HTL) from organic (i.e. sprio-MeOTAD) to polymer materials (i.e. PTAA)^2,4–6^. The critical problems of spiro-MeOTAD were reported with such as pin-holes, dopant diffusion, the vast intermixed region and complex fabrication^4–6^. The spiro-MeOTAD solution with Li salt and *t*-BP dopants attacks the hybrid perovskite thin film and makes the vast intermixed region with different chemical states affecting carrier mobility^4–6^. Especially, the creation of pinholes is the critical problem causing the infection to the main hybrid perovskite^7^. In the case of polymers for HTL, there are several merits, such as easy-fabrication and good hole carrier mobility^2,8–12^. For the OHP-based solar-cell device, however, it is not easy to find such kinds of polymer because of the weakness of hybrid perovskite thin films^5,13^. For instance, Poly(3-hexylthiophene-2,5-diyl) (P3HT) polymer, which is used for as HTL layers, showed the change in chemical states. (See the SFigs 1 and 2 in the Supplementary Material).

In our recent research, poly[4- (5-(4,8-bis(5-(6-((2-hexyldecyl)oxy)naphthalen-2-yl)thiophen-2-yl) benzo[1,2-b:4,5-b4,5-bearch, poly[4- (5-(4,8-bis(5-(6-((2-hexyldecyl)oxy)naphthalenbenzo[c][1,2,5]thiadiazole] (P1), poly[4-(5- (4,8-bis(5-(6-((2-hexyldecyl)oxy)naphthalen-2-yl)thiophen-2-yl) benzo[1,2-b:4,5-b1,2-b:4,5-b,2-b:4,5-b4,5-bstates.n-2-yl)-5-fluoro- 7-(4-octylthiophen-2-yl)benzo[c][1,2,5]thiadiazole] (P2) and poly[4- (5-(4,8-bis(5-(6-((2-hexyldecyl)oxy)naphthalen-2-yl)thiophen-2-yl) benzo[1,2-b:4,5-b1,2-b:4,5-b,2-b:4,5-b4,5-btes.n-2-yl)-5-fluoro- 7-(4-octylthiophen-2-yl)n ultra-benzo[c][1,2,5]thiadiazole] (P3) polymers for HTL materials with easy-fabrication showed a PCE_ave._ with 11.05–17.24% and a long lifetime (over 30 days) for the solar-cell device^14^. The thickness of P1, P2, and P3 polymers in this work was only 20 nm. For simplification, if we can use very thin HTL, the hole travel length will be short, and the hole mobility can be improved^8–12^. In this case, however, we should confirm the interface region. If an intermixed region at the interface is large, a short travel length is not the main factor for good mobility. Thus, understanding the chemical state of the interface between HTL and hybrid perovskite thin film is important before we try to understand hole carrier behavior in device characterization.

**Figure 1:**
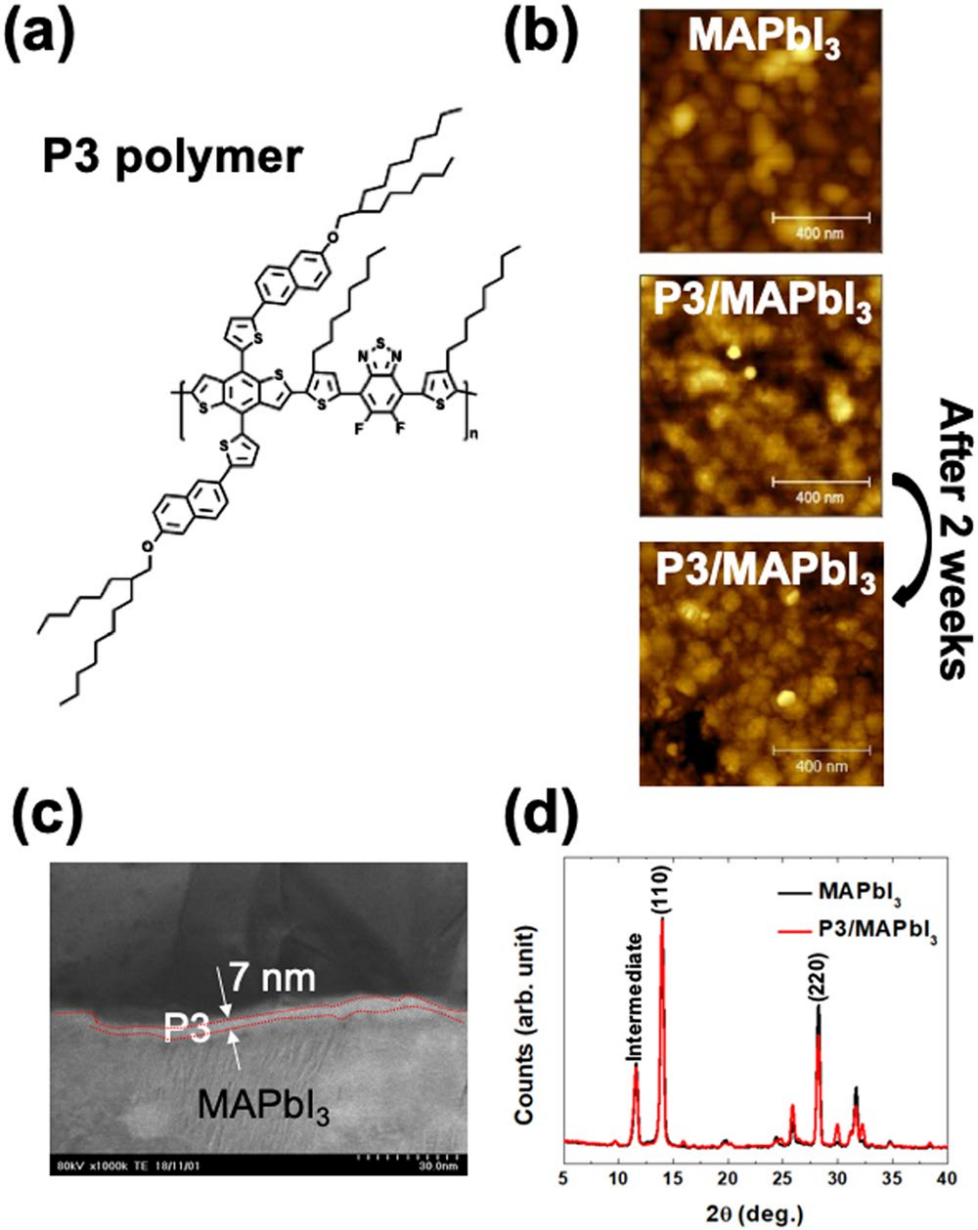
(**a**) The chemical formula of P3 polymer. (**b**) The surface morphologies of MAPbI_3_, P3/MAPbI_3_, and P3/MAPbI_3_ after 2 weeks on air. (**c**) The cross-sectional view using STEM. The thickness of P3 is about 7 nm. (**d**) XRD results of MAPbI_3_ and P3/MAPbI_3_. We could not observe any change significantly.

In this work, we fabricated CH_3_NH_3_PbI_3_ (MAPbI_3_) thin film using the sequential vacuum evaporation (SVE) method^15^ and then performed a spin-casting with the P3 polymer solution. (Fig. 1a) To confirm the intermixed region at the interface between the P3 polymer layer and the MAPbI_3_ thin film, we performed several measurements to visualize the physical properties, such as surface morphology, cross-sectional view of the P3/MAPbI_3_, atomic structure, and chemical states using atomic force microscopy (AFM), scanning transmission electron microscopy (STEM), x-ray diffraction (XRD), and x-ray photoelectron spectroscopy (XPS), respectively. To directly see the variable chemical states at the intermixed region, high-resolution XPS is one of powerful tool^11^. Finally, we confirmed the ultra-thin P3 polymer layer with 7 nm, and there was no significant intermixed state at the interface. After two weeks of air-exposure, we performed the same characterizations on the P3/MAPbI_3_ sample to evaluate air-stability.

## Results

To confirm the surface morphologies before and after the P3 polymer spin-casting, we performed AFM. (Fig. 1b) Before and after spin-casting, we could not observe any significant difference. However, the surface roughness changed from 14.2 to 11.4 nm. It appears that the P3 polymer does not affect the surface of the hybrid perovskite. After two weeks under air-exposure, the surface morphology of the P3/MAPbI_3_ sample did not change significantly. However, the surface roughness increased from 11.4 to 17.1 nm. We assume that this slight increase is due to the air-exposure.

From the STEM measurement, we can confirm the P3 polymer thickness was 7 nm. (Fig. 1c) Before and after spin-casting, the atomic structure of the MAPbI_3_ hybrid perovskite thin film obtained by XRD did not change. (Fig. 1d) The observed intermediate structure is due to the defect molecular-incorporated state fabricated by the SVE method, as previously reported^15^. From these results, we can confirm it is difficult to find an intermixed region in the measurement range of both STEM and XRD.

**Figure 2:**
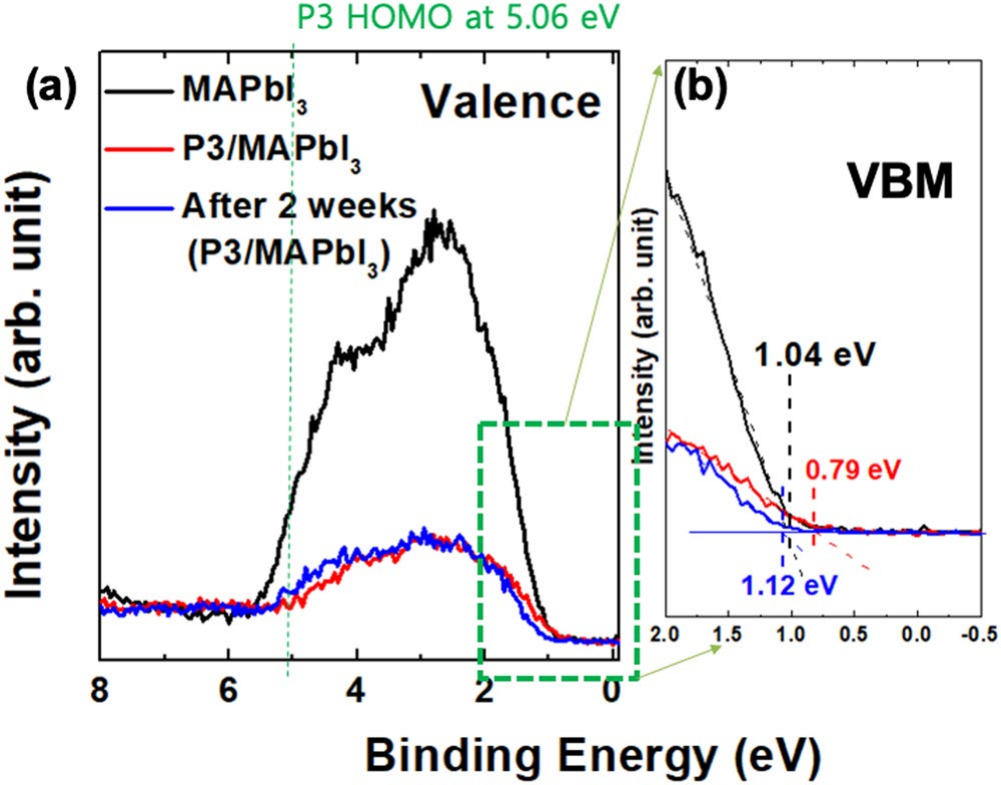
(**a**) The valence spectra of MAPbI_3_, P3/MAPbI_3_, and P3/MAPbI_3_ after 2 weeks on air. (**b**) The VBM spectra with 1.04, 0.79, and 1.12 eV of MAPbI_3_, P3/MAPbI_3_, and P3/MAPbI_3_ after 2 weeks on air, respectively.

To see the changes of chemical states from the surface (P3), interface (P3-MAPbI_3_), and bulk (MAPbI_3_), we performed high-resolution XPS. Before confirming all core-level spectra, we needed to perform an energy alignment based on each valence-band-maximum (VBM)^16^. (Fig. 2) From the high-occupied-molecular-orbital (HOMO) of P3 polymer with 5.06 eV, we assumed that all of the main valence band (Pb-I hybridization) originated mainly from the bulk MAPbI_3_ with a small contribution from the thin P3 polymer^17^. (Fig. 2a) From these valence bands, we obtained each VBM at 1.04, 0.79, and 1.12 eV from MAPbI_3_, P3/MAPbI_3_, and the after-two-weeks sample (P3/MAPbI_3_ on air-exposure during two weeks), respectively. (Fig. 2b) To calculate each chemical potential, we set the referenced VBM from the MAPbI_3_ thin film, and then obtained the chemical potentials with 0.25 and −0.08 eV of P3/MAPbI_3_ and the after-two-weeks sample, respectively.

**Figure 3:**
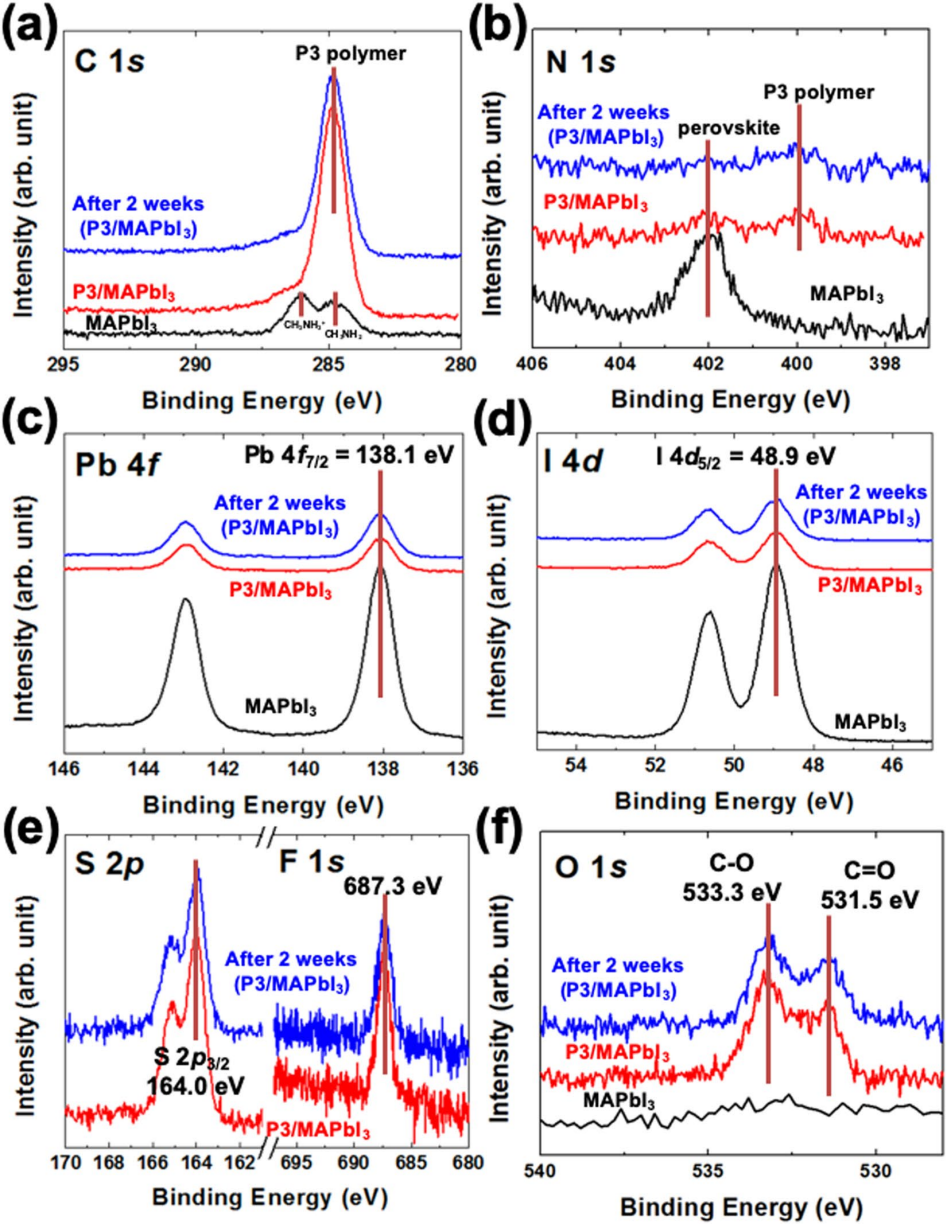
(**a**) C 1 *s*, (**b**) N 1 *s*, (**c**) Pb 4 *f*, (**d**) I 4*d*, (**e**) S 2*p*/F 1 *s*, and (**f**) O 1 *s* core-level spectra. Interestingly, there is no new chemical state originated from an interface.

All core-level spectra, C 1 *s*, N 1 *s*, Pb 4 *f*, I 4*d*, S 2*p*, F 1 *s*, and O 1 *s*, were calibrated with these obtained chemical potentials^16^. (Fig. 3) In the case of the MAPbI_3_ thin film, we can confirm the typical chemical states fabricated by the SVE method, such as the molecular defect-incorporated state, the single chemical state, and no oxygen element from C/N 1 *s*, Pb 4 *f*/I 4*d*, and O 1 *s* core-level spectra^15^. After spin-casting the P3 polymer, we found several significant results. A) We could not find any significant change before and after two weeks with air-exposure. (Fig. 3) From this finding, we can confirm that the P3 polymer with the ultra-thin layer (7 nm) has a good protection function for a hybrid perovskite thin film against water and oxygen contaminators from the air. B) The binding energies of Pb 4 *f* (4*f*_7/2_ = 138.1 eV) and I 4*d* (4*d*_5/2_ = 48.9 eV) originating from the bulk MAPbI_3_ were not changed before or after spin-casting the P3 polymer. (Fig. 3c,d) In the measurement range of high-resolution XPS, there was no creation of a vast intermixed region at the interface. It means that the P3 polymer layer makes a very clean interface between P3 polymer layer and hybrid perovskite thin film. That was the reason why we obtained the PCE of 17% from our previous report^14^. C) Lastly, we could observe the oxygen element in the two-weeks sample. (Fig. 3f) Consistently, the C 1 *s* core-level also changed. (Fig. 3a) However, we could not observe the change in chemical states of N 1 *s*, S 2*p* (2*p*_3/2_ = 164.0 eV), and F 1 *s* (1 *s* = 687.3 eV) core-level spectra. (Fig. 3b,e) (Also, there is only s single chemical state in S 2*p* and F 1 *s* core-levels without changing chemical states after two weeks of air exposure.) This means that the oxygen contaminations with C-O (533.3 eV) and C=O (531.5 eV) chemical states are incorporated with only carbon element on the surface of the P3 polymer^15^, and we can assume that there is no significant change with an intrinsic electrical property.

## Discussion

**Figure 4:**
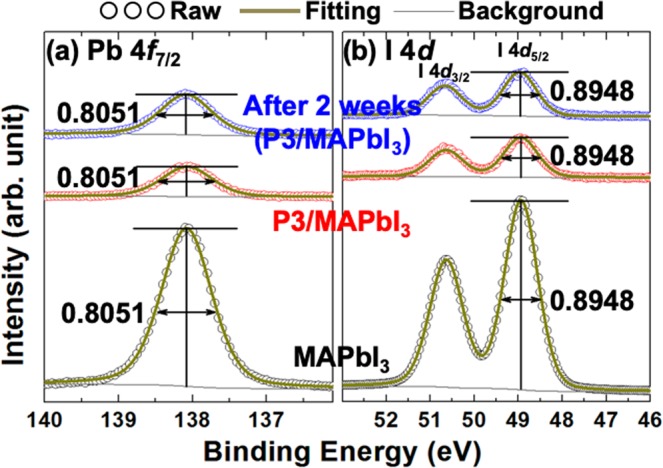
Curve-fittings of (**a**) Pb 4*f*_7/2_ and (**b**) I 4*d*_5/2_ core-level spectra. We found the same FWHM for each sample.

To see more details, we performed the curve-fittings on Pb 4*f*_7/2_ and I 4*d* core-level spectra. (Fig. 4) In the curve-fittings, we found the full-width-of-half-maximum (FWHM) with 0.8051 and 0.8948 eV in Pb 4*f*_7/2_ and I 4*d*_5/2_, respectively. (Fig. 4) Importantly, there is no change in FWHM in each spectrum. The intensity after spin-casting the P3 polymer decreased because of the different electron escape-depth^11^. However, we can observe a different chemical state or chemical shift in XPS measurement^11^. Basically, if we observe a different FWHM in the core-level spectra, it means a new chemical state or chemical shift^11^. In our measurements, interestingly, there is no change in FWHM in each spectrum, meaning that there is no new chemical state or chemical shift. This lack of change in FWHM is clear evidence of no formation of intermixed state at the interface between the P3 polymer layer with 7 nm and the MAPbI_3_ hybrid perovskite thin film. Additionally, there is no change in FWHM in the after-two-weeks sample. From this result, we can confirm that the P3 polymer with ultra-thin thickness (7 nm) can be a good protection layer for hybrid perovskite thin films.

In summary, we fabricated an ultra-thin P3 polymer layer (7 nm) for HTL of MAPbI_3_ hybrid perovskite and obtained the chemical states of the interface region between P3 and hybrid perovskite layers. The ultra-thin P3 polymer layer does not form a vast intermixed region at the interface. After exposing on the air for 2 weeks, the chemical states such as Pb 4 *f* and I 4*d* at the interface did not change. We confirmed that the P3 polymer layer is a proper protection layer for hybrid perovskite thin films against the air-exposure.

## Methods

### The sample preparation

OHP thin films were fabricated by the SVE method in a customized vacuum chamber^17^. *N*-type Si(100) and glass substrates were cleaned by 1) sonication in acetone (10 min), 2) rinsing in heated acetone (1 min), and 3) UV-Ozone treatment (30 min). The SVE chamber has two heaters for the evaporation of PbI_2_ (Lead(II) iodide, Sigma-Aldrich, 99% purity) and CH_3_NH_3_I (Methylammonium iodide, MAI, Sigma-Aldrich, 98% purity). The base pressure is 1.0 × 10^−2^ *Pa*. In the first step, the PbI_2_ with the deposition rate of 10 Å/s and the deposited thickness of 100 nm was evaporated onto the substrates at room temperature. For the second step, we evaporated the MAI with the fixed deposition rate and thickness of 2 Å/s and 280 nm^15^. We then prepared the P3 polymer solution with 10 mg P3 powder and 4 ml chlorobenzene. Finally, after the ultra-sonication for 20 min, we performed the spin-casting onto the formed MAPbI_3_ hybrid perovskite thin film with 4 krpm for 1 min.

### Thin film characterization

All formed OHP thin films were characterized by AFM (SPM-9700, Shimadzu), STEM (Hitachi HD-2700) with a focused ion beam (the ion source and image resolution are liquid gallium and 6 nm at 40 kV, respectively), XRD (RINT-TTRIII/NM with Cu*K*α source, Rigaku), and XPS (PHI5000 Versa ProbeII with a monochromated Al*K*α, ULVAC-PHI) to obtain the surface morphology, atomic structure, and chemical states, respectively.

### Curve-fittings of XPS core-level spectrum

For quantitative analysis, we performed the XPS curve-fitting. (Fig. 4) We fitted Pb 4*f*_7/2_ and I 4*d* core-level spectra using Doniach-Sŭnjić curves convoluted with a Gaussian distribution of 0.5 eV FWHM^17^. Background due to inelastic scattering was subtracted by the Shirley (integral) method.

## Supplementary information

The interface chemical states in P3HT/MAPbI3
