# Supplementary material for: Clean interface without any intermixed state between ultra-thin P3 polymer and CH3NH3PbI3 hybrid perovskite thin film
Source: Sci Rep. 2019 Jul 26;9:10853. doi: 10.1038/s41598-019-47252-y (PMC6659629; doi:10.1038/s41598-019-47252-y)

Supplemental Information

Title

Clean Interface without any intermixed state between ultra-thin P3 polymer and $CH_3NH_3PbI_3$ hybrid perovskite thin film

Authors

Min-Cherl Jung[1,*], Asuka Matsuyama[1], Sora Kobori[1], Inhee Maeng[2], Young Mi Lee[3], Myungkwan Song[4], Sung-Ho Jin[5], and Masakazu Nakamura[1]

Affiliations

[1]Division of Materials Science, Nara Institute of Science and Technology, Ikoma, Nara, 630-0192, Japan

[2]Advanced Photonics Research Institute, Gwangju Institute of Science and Technology, Gwangju, 61005, Republic of Korea

[3]Beamline department, Pohang Accelerator Laboratory, POSTECH, Pohang, 37673, Republic of Korea

[4]Surface Technology Division, Korea Institute of Materials Science (KIMS), Changwon, Gyeongnam, 642-831, Republic of Korea

[5]Department of Chemistry Education Graduate, Department of Chemical Materials, and Institute for Plastic Information and Energy Materials, Pusan National University, Busan 46241, Republic of Korea

[*]Correspondence to: mcjung@ms.naist.jp

Method

The sample preparation. We prepared the P3HT polymer solution with 10 mg P3HT powder and 4 ml chlorobenzene. After the ultra-sonication for 20 min, we performed the spin-casting onto the formed $MAPbI_3$ hybrid perovskite thin film with 4 krpm for 1 min.

Fig. S1. Valence spectra of $MAPbI_3$ and P3HT/$MAPbI_3$ thin films. The valence spectrum was changed after the P3HT spin-casting.

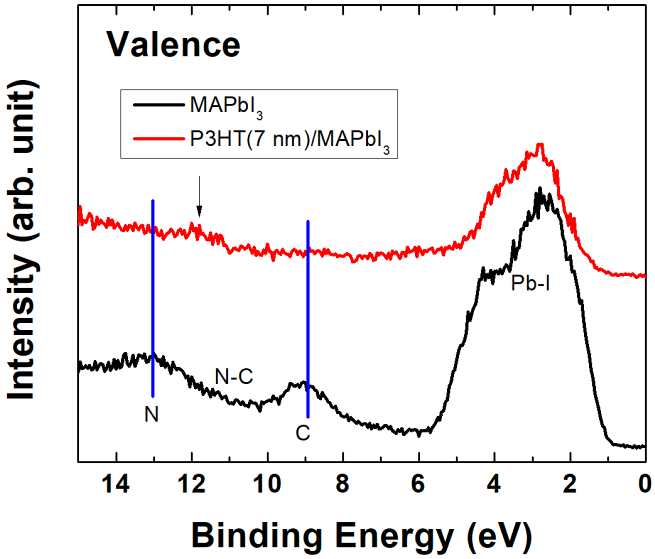

Fig. S2. Pb 4*f* and I 4*d* core-level spectra of $MAPbI_3$ (black) and P3HT/$MAPbI_3$ (red) thin films. After the P3HT spin-casting, 1) The maximum peak position is shifted to the low binding energy in Pb 4*f* core-level spectrum. And 2) the new chemical state appears at the low binding energy in I 4*d* core-level spectrum.

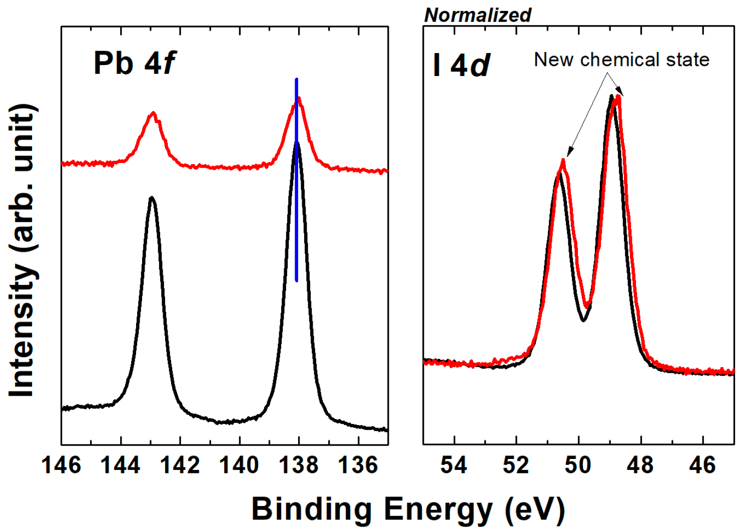

Supplement: Supplementary file 1 — The interface chemical states in P3HT/MAPbI3 [file 41598_2019_47252_MOESM1_ESM.pdf]
